# Response of the chemical structure of soil organic carbon to modes of maize straw return

**DOI:** 10.1038/s41598-021-84697-6

**Published:** 2021-03-22

**Authors:** Shuqing Zheng, Jiuming Zhang, Fengqin Chi, Baoku Zhou, Dan Wei, Enjun Kuang, Yu Jiang, Gang Mi, Yu ping Chen

**Affiliations:** 1 Heihe Branch Academy of Heilongjiang Academy of Agricultural Sciences, Heihe, 164300 China; 2 Key Laboratory of Soil Environment and Plant Nutrition of Harbin, Institute of Soil and Fertilizer and Environment Resources, Heilongjiang Academy of Agricultural Sciences, Harbin, 150086 China; 3 Heilongjiang Academy of Agricultural Sciences Postdoctoral Program, Institute of Soil and Fertilizer and Environment Resources, Heilongjiang Academy of Agricultural Sciences, Harbin, 150086 China; 4 Institute of Plant Nutrition and Resources, Beijing Academy of Agriculture and Forestry Sciences, Beijing, 100097 China

**Keywords:** Agroecology, Carbon cycle

## Abstract

Elucidating the chemical structure of soil organic matter (SOM) is important for accurately evaluating the stability and function of SOM. Aboveground vegetation directly affects the quantity and quality of exogenous organic matter input into the soil through plant residues and root exudates, which in turn affects soil microbial species, community structure, and activity, and ultimately impacts the chemical structure of SOM. In this study, a ^13^C nuclear magnetic resonance technique was used to analyze the chemical structure characteristics of soil organic carbon (SOC) under various rates of straw returning combined with rotary tillage and under full straw mulching. The results showed that full straw returning with rotary tillage and full straw mulching more effectively increased the SOC content than reduced rate of straw returning (1/2 and 1/3 of full straw) with rotary tillage. The contents of alkyl C and alkoxy C in the functional groups of SOC under various straw returning treatments were increased compared with those under the treatment of maize stubble remaining in soil (CK). Furthermore, the contents of aromatic C and carboxyl C were decreased, which were consistent with the chemical shift changes of SOC. Compared with CK treatment, straw returning decreased the content of aromatic C in the functional groups of SOC, but increased the content of alkoxy C, which could be associated with the change in integral areas of absorption peaks of alkyl C and alkoxy C moving toward left and right, respectively. The content of total SOC was significantly positively (*P* < 0.05) correlated with that of alkoxy C and significantly negatively (*P* < 0.01) correlated with that of aromatic C. The molecular structure of SOC tends to be simplified due to the decreasing in refractory C and the increasing in easily decomposed C after straw returning to the field.

## Introduction

Elucidating the chemical structure and stability of soil organic matter (SOM) is key to understanding soil fertility and carbon (C) cycling. ^13^C nuclear magnetic resonance (^13^C-NMR) spectroscopy is an advanced technology for determining the chemical structure of SOM. It can simultaneously perform qualitative and quantitative analysis for the chemical composition of SOM and is thus an effective means of studying the chemical structure and transformation of SOM^1^. Differences in the composition of plant residues reflect the differences in SOM under continuous degradation of residues, which is the primary factor accounting for the variation in the chemical structure of soil organic carbon (SOC) under different land use types^2^. The chemical structure and stability of SOC are closely related to the types of aboveground vegetation and the quantity and quality (chemical structure) of exogenous organic matter entering soil. Clemente et al.^3^ found that the chemical structure of SOC was changed with the decomposition of the addition materials from different parts of maize plant (roots, stems, and leaves) to soil. A high carbohydrate content is found in SOC under the addition of maize stem, and a high aliphatic C content is recorded in the soil humus under the addition of maize leaf. Maize leaf tissue has a high aliphatic C content, which contributes to the stability of SOC. Increasing the input of exogenous organic matter is an important measure for maintaining and enhancing SOC and improving soil structure.

Straw returning to the soil not only has the potential to alleviate soil degradation, but also increases the sequestration potential of SOC. Therefore, studying the effects of continuous maize straw returning and tillage modes as well as the amount of returning straw on the chemical structure of SOC is significant for reducing soil degradation and improving soil quality.

## Results

### Changes in soil organic carbon content under different straw returning measures

**Figure 1:**
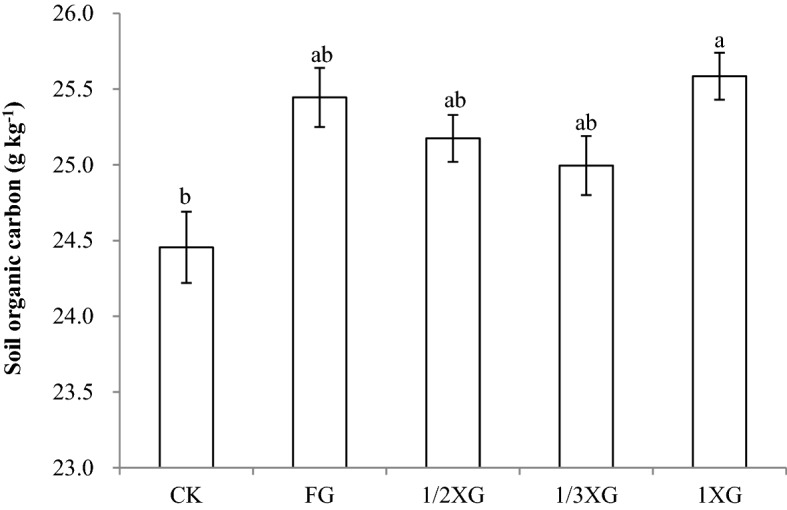
Effect of straw returning on soil organic carbon content. *CK* control (stubble remaining in soil), *FG* full straw mulching, *1 XG* full straw returning combined with rotary tillage, *1/2 XG* 1/2 of full straw returning combined with rotary tillage, *1/3 XG* 1/3 of full straw returning combined with rotary tillage.

It can be seen from Fig. 1 that the highest content of SOC (25.6 g kg^−1^) was found in the treatment of full straw returning combined with rotatory tillage (1XG), and the lowest content of SOC (24.5 g kg^−1^) was found in the treatment of stubble remaining in soil (CK). The difference in SOC was significant (*P* < 0.05) between these treatments. The SOC content in the full straw mulching treatment was 25.4 g kg^−1^, which was higher than that in the CK treatment and in the treatments of reduced rate of straw returning combined with rotary tillage (1/2 XG and 1/3 XG), but this difference was not statistically significant (*P* > 0.05). The above results showed that long-term straw return could impact the soil organic carbon content. Of the treatments, full rotary tillage return performed better than reduced-rate rotary tillage return and straw mulching return.

### CPMAS^13^C-NMR spectral characteristics of soil organic carbon with different straw returning measures

**Figure 2:**
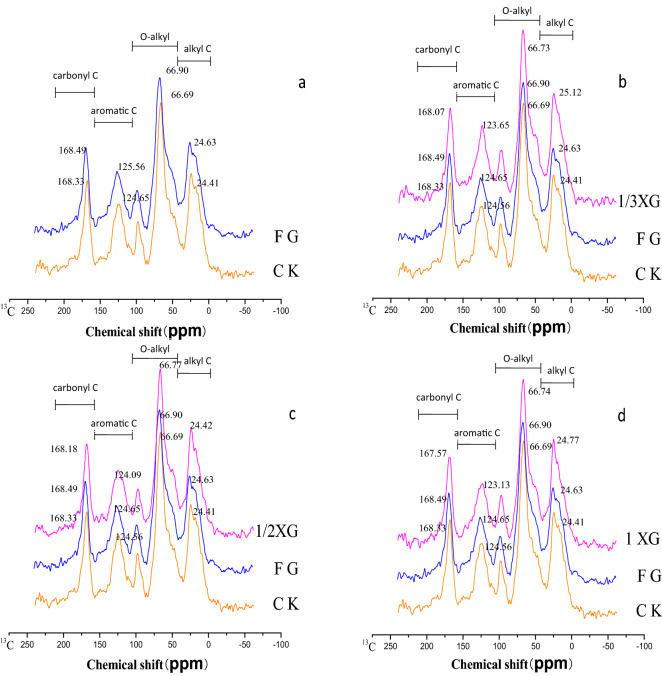
CPMAS ^13^C-NMR spectra of organic carbon in Black soil treated with different straw returning treatments. *CK* control (stubble remaining in soil), *FG* full straw mulching, *1 XG* full straw returning combined with rotary tillage, *1/2 XG* 1/2 of full straw returning combined with rotary tillage, *1/3 XG* 1/3 of full straw returning combined with rotary tillage. (**a**) The comparison of FG with CK; (**b**) the comparison among 1/3 XG, FG, and CK; (**c**) is the comparison among 1/2 XG, FG, and CK; (**d**) is the comparison among XG, FG, and CK.

As shown in Fig. 2, the absorption peak of alkyl C in SOC in the different straw returning treatments was around 24 ppm, which represented the chemical shift of the methyl C (CH_3_) in long-chain aliphatic compounds, wax, and cutin. The absorption peaks of alkoxy C were mainly around 66 ppm and 97 ppm, which were assigned to the alkyl C of carbohydrate alcohols, amino sugars, and the di-O-alkyl C of hemicellulose, respectively. The absorption peak of aromatic C was mainly around 122–125 ppm, mainly corresponding to the phenyl ring C (Aryl C) of tannin and lignin. In the carbonyl C region, the main signal appeared near 168 ppm, which was the absorption of carboxylic acid, ester, and amide C^4^.

In the alkyl C region, the chemical shifts of the absorption peaks of SOC functional groups were increased in all straw returning treatments compared with those in the CK treatment. The chemical shift of the absorption peaks in the 1/3 XG treatment was 25.12 ppm, which was higher than that in the FG treatment, whereas the chemical shifts of the absorption peaks in 1/2 XG and 1 XG treatments were lower than that in the FG treatment. In the alkoxy C region, the chemical shifts of the absorption peaks of SOC functional groups in all straw returning treatments were increased compared with those in the CK treatment, and the chemical shifts in all straw returning combined with rotary tillage treatments were lower than that in the FG treatment. In the aromatic C region, the chemical shifts of the absorption peaks in all straw returning combined with rotary tillage treatments were lower than that in the CK treatment, and the smallest chemical shift of the absorption peak (123.13 ppm) was found in the 1 XG treatment. In the carboxyl C region, the chemical shift of the absorption peak in straw returning combined with rotary tillage treatments (1/3 XG, 1/2 XG, and 1 XG) was lower than that in the CK treatment; and similarly, the lowest chemical shift of the absorption peak (167.57 ppm) was found in the full straw returning combined with rotary tillage treatment (Fig. 2b–d). Compared with FG treatment, all straw returning combined with rotary tillage treatments increased the chemical shifts of the absorption peak of the alkyl C, and decreased those of alkoxy C, aromatic C, and carboxyl C of SOC.

### Changes in the chemical structure of soil organic carbon under different straw returning measures

Compared with that of CK, all straw returning combined with rotary tillage treatments increased the contents of alkyl C and alkoxy C in the SOC functional groups, but decreased the contents of aromatic C and carboxyl C (Table 1). The highest content of alkyl C (25.2%) was found in the 1/2 XG treatment. The alkoxy C contents in the 1/2 XG and 1 XG treatments were 2.6% higher significantly (*P* < 0.05) than that in the CK treatments. The higher contents of aliphatic C (alkyl C + alkoxy C) of SOC was found in the 1/2 XG and in the 1 XG treatments compared with CK treatment. The FG treatment and 1 XG treatment exhibited a relatively large (*P* < 0.05) decrease in the content of aromatic C than CK treatment. The 1/2 XG and 1/3 XG treatments showed a relatively large (*P* < 0.05) decrease in the carboxyl C content than CK.

**Table 1:** Relative proportions of functional groups with different chemical shift intervals in the CPMAS^13^C-NMR spectra of black soil organic carbons from different straw return treatments.

Treatment	Alkyl C (%)	O-alkyl C (%)	Aromatic C (%)	Carbonyl C (%)	Aliphatic C/Aromatic C	Alkyl C/O-alkyl C	Hydrophobic C/Hydrophilic C
CK	24.2 ± 0.12a	38.3 ± 0.39b	19.8 ± 0.45a	17.8 ± 0.42a	3.16 ± 0.02b	0.63 ± 0.03a	0.78 ± 0.02a
FG	24.9 ± 0.19a	39.1 ± 0.36ab	18.7 ± 0.72b	17.4 ± 0.56a	3.43 ± 0.03a	0.64 ± 0.03a	0.77 ± 0.03a
1/3 XG	24.8 ± 0.11a	38.9 ± 0.55b	19.5 ± 0.63ab	16.8 ± 0.38b	3.26 ± 0.01a	0.64 ± 0.05a	0.79 ± 0.02a
1/2 XG	25.2 ± 0.10a	39.3 ± 0.42a	19.1 ± 0.40ab	16.4 ± 0.34b	3.37 ± 0.02a	0.64 ± 0.06a	0.80 ± 0.04a
1 XG	24.7 ± 0.10a	39.3 ± 0.43a	18.5 ± 0.31b	17.5 ± 0.30a	3.45 ± 0.02a	0.63 ± 0.03a	0.76 ± 0.03a

*Aliphatic C/Aromatic C* (Alkyl C + O-alkyl C)/Aromatic C, *Hydrophobic C/Hydrophilic C* (Alkyl C + Aromatic C)/(O-alkyl C + Carbonyl C), *CK* control (i.e., stubble remaining), *FG* full straw mulching return, *1 XG* full rotary tillage return, *1/2 XG* 1/2 rotary tillage return, *1/3 XG* 1/3 rotary tillage return. Different letters in the same column represent significant difference (*p* < 0.05).

The ratio of aliphatic C content to aromatic C content (aliphatic C/aromatic C) was higher in the straw returning treatments than that in the CK treatment. The ratios of alkyl C content to alkoxy C content and hydrophobic C content to hydrophilic C content were not significantly different among each treatment.

### Relationship between soil organic carbon and the chemical structure of soil organic carbon

**Figure 3:**
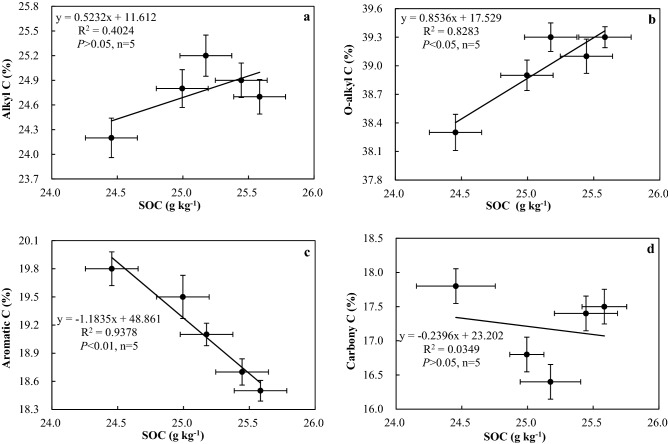
Correlation between soil organic carbon content, clay content, and relative proportion of organic carbon functional groups.

The total SOC had a positive correlation with the contents of alkyl C and alkoxy C and a negative correlation with the contents of aromatic C and carboxyl C (Fig. 3). The total SOC content was significantly positively (*P* < 0.05) correlated with the content of alkoxy C and was significantly negatively (*P* < 0.01) correlated with the content of aromatic C (Fig. 3b,c). The increase in SOC content was more favorable for decreasing the aromatic C content and increasing the alkoxy C content.

## Discussion

Straw returning improves soil structure and increase soil nutrients, while the mode and amount of straw returning to the field could inevitably affect soil structure and nutrient status. In this study, full straw returning combined with rotary tillage and full straw mulching more effectively increased the SOC content.

Compared with CK, each straw returning treatment increased the contents of alkyl C and alkoxy C of SOC functional groups and decreased the contents of aromatic C and carboxyl C, which was consistent with the chemical shift changes of the absorption peaks of SOC functional groups in each straw returning treatment. The abundance peaks of alkyl C and alkoxy C moved toward left and right in straw returning treatment, respectively, which leads to the change in integral area of their abundance peaks. The alkyl C content of the soil organic carbon in each straw return treatment increased compared with that in the CK treatment, but the difference between the treatments was not significant. At the same time, the content of alkoxy C increased significantly, and the full rotary tillage return treatment exhibited the most significant drop in aromatic C content compared with that in CK (*P* < 0.05). The results indicated that the content of soil refractory carbon decreased following straw return, and the content of the carbon that is easily metabolized and utilized by microorganisms increased, with the result that the full rotary tillage return treatment was better than the full straw mulching return treatment. Straw addition to soil provides C and N sources for microorganism. About 8–35% of straw C can be preserved in soil as organic matter^5^. Microorganisms play important roles in soil C cycling, in turn, their metabolic products are the main components of SOC ^6^. Lehmann et al. suggested that the stable SOC partly originates from microbial re-synthese or biomass re-cycling^7^. Further research should be combined microbial stabilized mechanism of SOC with chemical structure of SOC. The ratio of aliphatic C/aromatic C can reflect the complexity of the molecular structure of humic substances. A higher ratio indicates a lower content of aromatic core structures in humic substances, more aliphatic side chains, a lower degree of condensation, and a simpler molecular structure ^8^. The results of this study demonstrated that the ratio of aliphatic C/aromatic C of the soil organic carbon in the full rotary tillage return treatment was higher than in the other treatments. The above results also further confirmed that with the continuous degradation of the residues, the differences in the composition of plant residues were ultimately converted into differences in the structure and composition of the soil organic matter^2^.

Most organic matter in the soil is important for the dynamics of soil organic matter. Alkyl C in SOC functional groups mainly originates from carbohydrate easily decomposed by soil microorganism, including hemicellulose, cellulose, polymeric and non-polymeric carbohydrates, and ethanol-like substance^9^. It means that alkyl C is belonged to labile organic C. Ratio of alkyl C to alkoxy C is a sensitive index for SOC decomposition degree, and can be used to evaluate the degree of alkylation of humic substance^10^. The lower ratio of alkyl C to alkoxy C indicates lower decomposed degree of soil organic matter. Hydrophobic alkyl-C plays an essential basic role in the accumulation and sequestration of SOC in forest and cultivated soils^11^. Aromatic C is mainly derived from organic compound with benzene ring, such as lignin, suberin, polypeptide and black C, or is originated from microbial metabolites or charred organic matter, suggesting that aromatic C is belonged to recalcitrant organic C^12^. Mathers and Xu found that alkoxyl C is the easily decomposed functional group in plant residue^13^. During the initial decomposed period of plant residues, alkoxy C in them is rapidly decomposed and, on the contrary, aromatic and alkyl C is selectively stabilized in soil. Mahieu et al.^14^ showed that the soil with a higher organic matter content has a higher ratio of alkoxy C. The large amount of straw addition to soil could promote their decomposition in soil, which could lead to the instability of soil organic matter, and further being beneficial for the release of soil nutrients and for increasing crop yields. The correlation analysis results in this paper showed that the increase in SOC content was more favorable for increasing alkoxy C content and reducing the aromatic C content. The results also clarified (Table 1) the response of straw returning measures to the chemical structure of SOC. The organic C stabilization in soil is associated with their physical protection by soil microaggregates and oxyhydrates^15^, or with the recalcitrant matter protected by hydrophobic organic matter^16^, such as lignin and lipids^12^. Figure 1 showed that straw returning increased the contents of total SOC relative to CK treatment. Table 1 showed that straw returning increased the contents of aliphatic C compared with CK treatment. These data indicated that the selective immobilization and retention of aliphatic molecules such as hydroxyl acids, fatty acids and alkanes leads to the accumulation of SOC^17,18^.

## Conclusions

The full straw returning combined with rotary tillage significantly increased the content of soil organic carbon compared with the modes of reduced rate of straw returning and full straw mulching. Compared with the treatment of stubble remaining in soil, the long-term straw returning treatments increased the contents of alkyl C and alkoxy C of soil organic carbon functional groups, and decreased the contents of aromatic C and carboxyl C, which were consistent with the chemical shifts of the absorption peak of SOC functional groups in straw returning treatments. All the results indicated that after straw returning to the field, the content of refractory carbon in the soil decreased, the content of easily decomposed carbon increased, and the molecular structure was simplified.

## Methods

### Experimental design

This experiment was conducted at the Science and Technology experimental site (125° 27′ 5″ N, 49° 33′ 35″ E) of the North Corporation of Sinograin, Nenjiang, Heilongjiang, China. The soil in the tested area was classified as Black soil according to Chinese Soil Taxonomy (as Mollisol according to USDA classification system) with a thick humus layer and clay texture. The area has a mid-temperate continental monsoon climate with an average annual temperature of − 1.4 to 0.8 °C, precipitation of 450 mm, a frost-free period of 115 days, and an effective accumulated temperature of 2150 °C. The basic physicochemical properties at 0–20 cm plow layer of soil were shown in Table 2. The experiment included two modes: maize straw mulching (FG) and straw returning combined with rotary tillage. In the test plot, there were six 10-m ridges per treatment, and each treatment area was 39 m^2^. Each treatment has three replicate. The experiment started in 2012 under the continuous planting of maize, and the straw was mechanically crushed and returned to the field in the fall. In accordance with the adjustment in the C/N of the straw, the amount of applied fertilizer was N 150 kg hm^−2^, P_2_O_5_ 75 kg hm^−2^, and K_2_O 75 kg hm^−2^. Five treatments were included: (1) stubble remaining in the field, which served as the control (CK); (2) full straw mulching (FG); (3) full straw returning combined with rotary tillage (1 XG) ; (4) 1/3 of full straw returning combined with rotary tillage (1/3 XG); and (5) half of full straw returning combined with rotary tillage (1/2 XG).

**Table 2:** Basic physicochemical properties of surface soil.

Item	Value
Organic matter (g kg^−1^)	45.9
Total N (g kg^−1^)	2.5
Total P (g kg^−1^)	2.0
Total K (g kg^−1^)	22.7
Available N (mg kg^−1^)	211.9
Available P (mg kg^−1^)	78.6
Available K (mg kg^−1^)	226.7
Clay content (< 0.002 mm, g kg^−1^)	156.6
Silt content (0.002 ~ 0.02 mm, g kg^−1^)	657.7
Sand content (> 0.02 mm, g kg^−1^)	185.7

### Sample collection

Soil samples were collected after the maize (Demeiya 2) was harvested in November 2019. Five soil cores (diameter 5 cm) were randomly taken from 0 to 20 cm depth in each plot, mixed thoroughly, and packed into cloth bags. After the crop roots and other debris were removed, the samples were air-dried for analyzing the content and chemical structure of SOC.

### Determination of total soil organic carbon

The content of total SOC was measured using a TOC (total organic carbon) analyzer (NC2100, Jena, Germany,) after air-dried soil samples were passed through a 100-mesh sieve.

### Purification of soil organic carbon

Five grams of air-dried soil was added to a 100 mL plastic centrifuge tube, followed by the addition of 50 mL of hydrogen fluoride (HF) solution (10% v/v). After the tube was capped, the solution was shaken for 1 h and centrifuged for 10 min (3000 r min^−1^), and the supernatant was removed. Subsequently, the residue in the tube was treated with HF solution and then followed the above shaking and centrifuging steps. A total of eight repeats (according to the conditions of the actual samples) were performed with different duration of shaking (4 × 1 h, 3 × 12 h, and 1 × 24 h). Lastly, the residue in the tube was washed with double-distilled water four times, mainly to remove the residual HF in the soil sample. The detailed steps were as follows: 50 mL of double-distilled water was added into tube, shaken for 10 min and centrifuged (3000 r min^−1^) for 10 min, and then the supernatant was removed. The purified samples with free-HF were dried in an oven at 40 °C, ground through a 60-mesh sieve, and stored in a Zip-lock bag for NMR measurement.

### Determination of the chemical structure of soil organic carbon

The ^13^C solid-state NMR spectrum was collected on a Bruker AV400 NMR spectrometer (Switzerland). The cross-polarization magic-angle spinning (CPMAS) technique was used, the ^13^C NMR frequency was 400.18 MHz, the magic angle spinning frequency was 8 kHz, the contact time was 2 ms, the delay time was 3 s, and the number of data points was 3000. The chemical shift was calibrated based on the external standard sodium 2, 2-dimethyl-2-silapentane-5-sulfonate (DSS), the integrated area was automatically given by the instrument, and the relative content of organic C in each functional group of SOC was expressed as the percentage of the integrated area of a chemical shift interval to the total integrated area. The C structures corresponding to the chemical shift of the main ^13^C signal of SOC (Table 3) were as follows: alkyl C region (0–45 ppm), alkoxy C region (45–110 ppm), aromatic C region (110–160 ppm), and carbonyl C region (160–220 ppm)^4,19^.

**Table 3:** ^13^C solid-state NMR determination of organic carbon functional groups and corresponding high-molecular-weight compounds.

Chemical shift	Functional groups of organic matter		Macro-molecular compounds
0–45	Nonpolar alkyl C	CH_3_	Long chain, aliphatic, waxes, cutin, suberin
		(CH_2_)n/C&CH	
45–60	O-Alkyl C	Methoxyl C	N-Alkyl, amino acids, lignin
60–93		Carbonhydrate	Alcohols, amino sugars, tariric acid, dehydromatricaria ester, polyynes
93–110		di-O-alkyl C	Hemicelluloses, cellulose,
110–142	Aromatic C	Aryl C	Tannin, lignin
142–160		Phenolic	Tannin, lignin, suberin
160–190	Carbonyl C	Carboxy C, Amide C	Amide, ester
190–220		Carbonyl C	Ketone, quinine, aldehyde

### Data analysis

NMR spectra (CPMAS ^13^C-NMR) were analyzed using MestReNova professional software. After analyzing and extracting the source data, Microsoft Office Excel 2010 and Origin 8.0 software were used for data processing and plotting. The data in the “Available Data” were plotted using Origin by overlapping the fitted curve, and SPSS 17.0 (SPSS Inc., Chicago, USA) statistical analysis software was used to test for significant differences (Duncan’s method) and for correlation analysis.
